# Supplementary material for: On the Hydration of Heavy Rare Earth Ions: Ho3+, Er3+, Tm3+, Yb3+ and Lu3+—A Raman Study
Source: Molecules. 2019 May 21;24(10):1953. doi: 10.3390/molecules24101953 (PMC6572539; doi:10.3390/molecules24101953)
Supplement: Supplementary file 1 [file molecules-24-01953-s001.pdf]

Supplementary Materials

Table S1. Band fit data on of the anisotropic spectrum of an aqueous 2.423 molL^{-1} $Yb(ClO_4)_3$ solution in the terahertz region. Five YbO_8 skeleton modes of $[Yb(H_2O)_8]^{3+}$(aq) were detected. In addition to isotropic band at 390 cm^{-1} (fwhh = 59 cm^{-1}) was observed with an integrated band intensity = 3160.

$[Yb(H_2O)_8]^{3+}$		
peak position / cm^{-1}	Integrated band area	fwhh / cm^{-1}
88.5	941	119
158.7	782	97.6
229.4	752	75.7
260.0	278.6	68
333.2	677	85.3

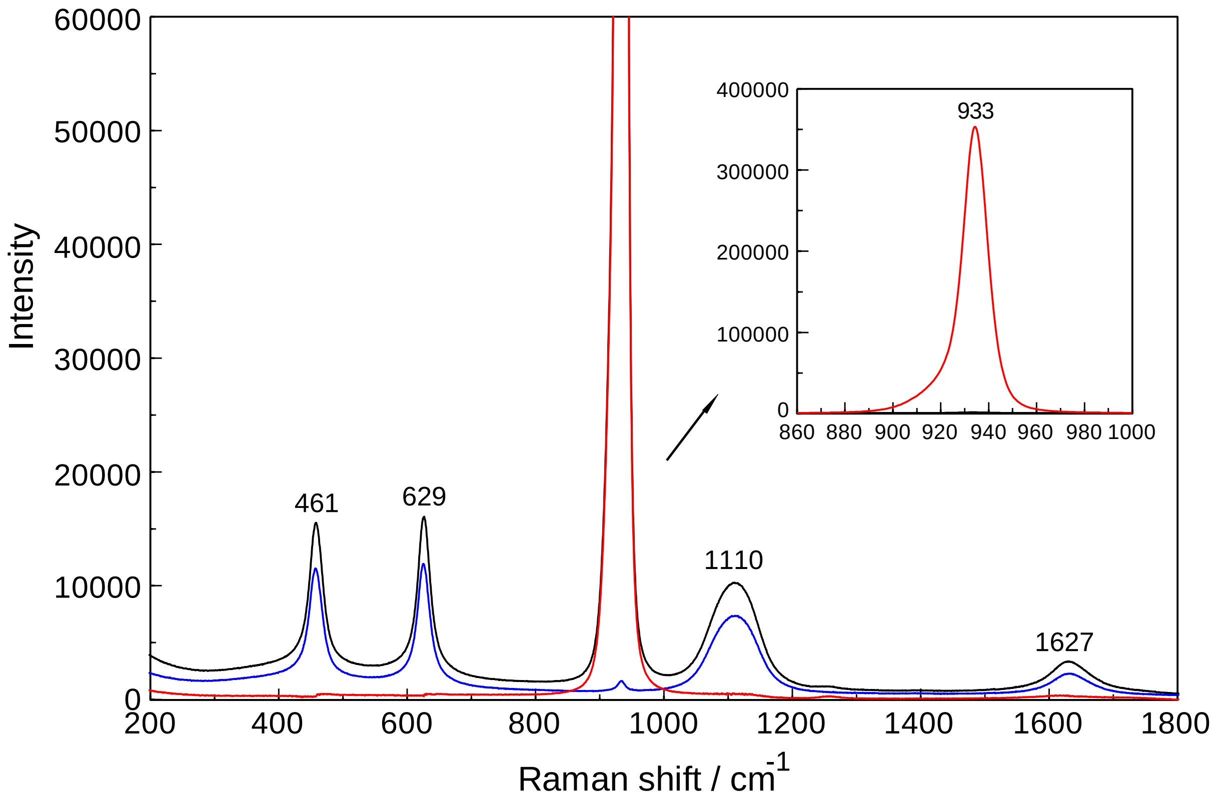

Figure S1. Raman scattering profiles (I_{VV} (black), I_{VH} (blue) and I_{iso} (red)) of a 3.800 molL^{-1} $NaClO_4$(aq) solution. The depolarized modes, ν_2(e) at 461 cm^{-1} and $\nu_2(f_2)$ at 629 cm^{-1} are the deformation modes of perchlorate, ClO_4^-(aq),.The strongly polarized mode, $\nu_1(a_1)$ at 933 cm^{-1} is the Cl-O symmetric stretching mode and the depolarized band, $\nu_3(f_2)$ at 1110 cm^{-1} is due to the antisymmetric stretching mode of ClO_4^-(aq). The broad mode at 1634 cm^{-1} is due to the deformation mode of H_2O.

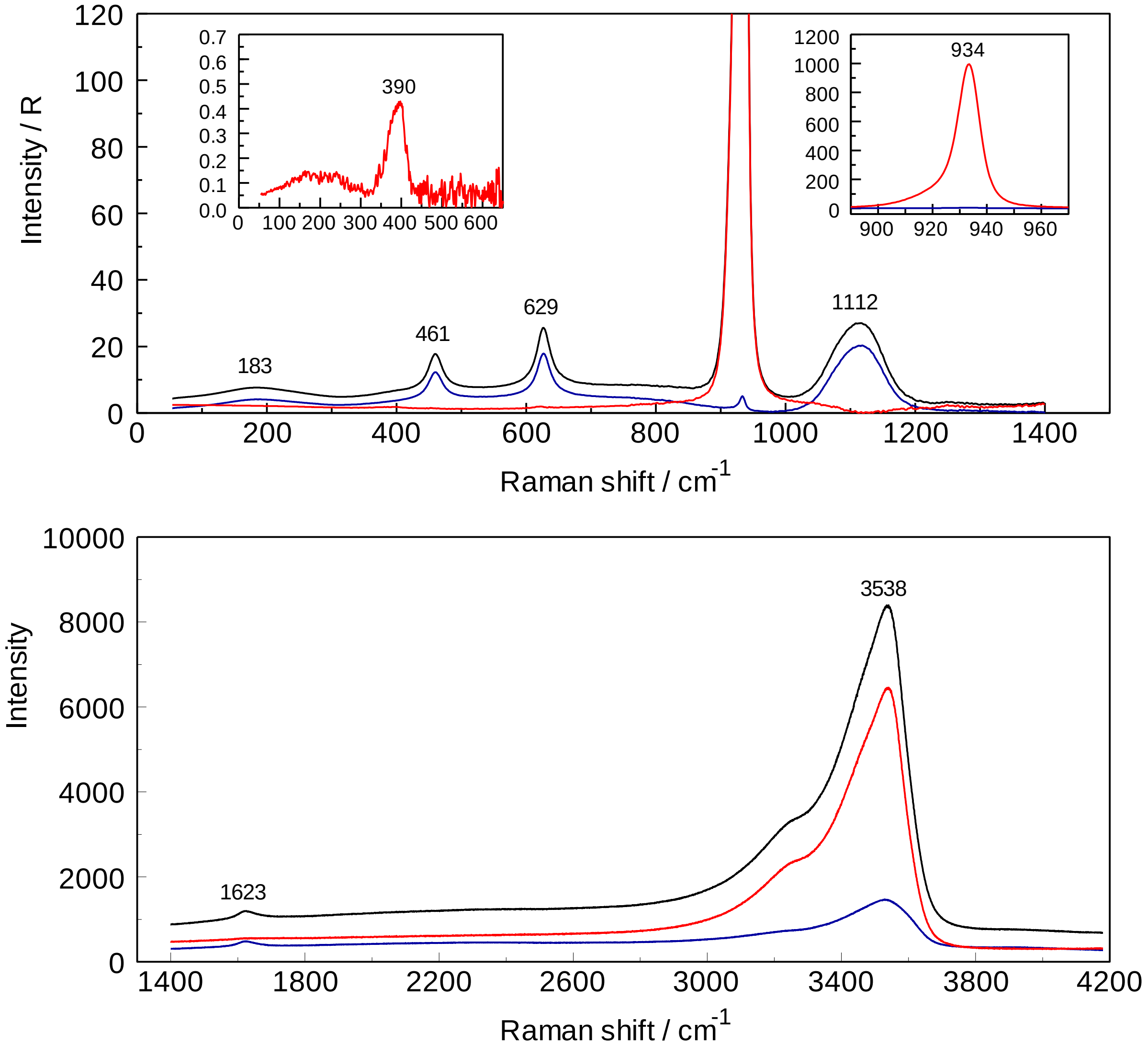

Figure S2. Top panel: Raman scattering profiles (R_{VV}, R_{VH} and R_{iso}) of a 2.423 molL^{-1} $Yb(ClO_4)_3$ solutions in water. The left inset shows the symmetric YbO_8 stretching mode of $[Yb(H_2O)_8]^{3+}$ at 390 cm^{-1}. Note the perchlorate bands at 461 cm^{-1} and at 629 cm^{-1} as well as the band at 1112 cm^{-1}. The inset at the right shows the $\nu_1ClO_4^-$ mode at its full scale. Shown are the isotropic scattering profile (red), the anisotropic one (blue) and the polarized scattering (black).

Bottom panel: Raman spectrum of the 2.423 molL^{-1} $Yb(ClO_4)_3$ solution in the wavenumber region from 1400 cm^{-1} to 4160 cm^{-1} (spectra in I-format: from top to bottom: polarized (black), depolarized (blue) and isotropic (red) scattering profiles). The deformation mode of water appears at 1623 cm^{-1} and in the O-H stretching region the water band is modified by the typical ClO_4^- H_2O mode of weakly hydrogen bonded O-H oscillators. The band at 3538 ± 3 cm^{-1} is due to the stretching bands of the weakly bonded O-H····$OClO_3^-$ units of H_2O/ClO_4^-.

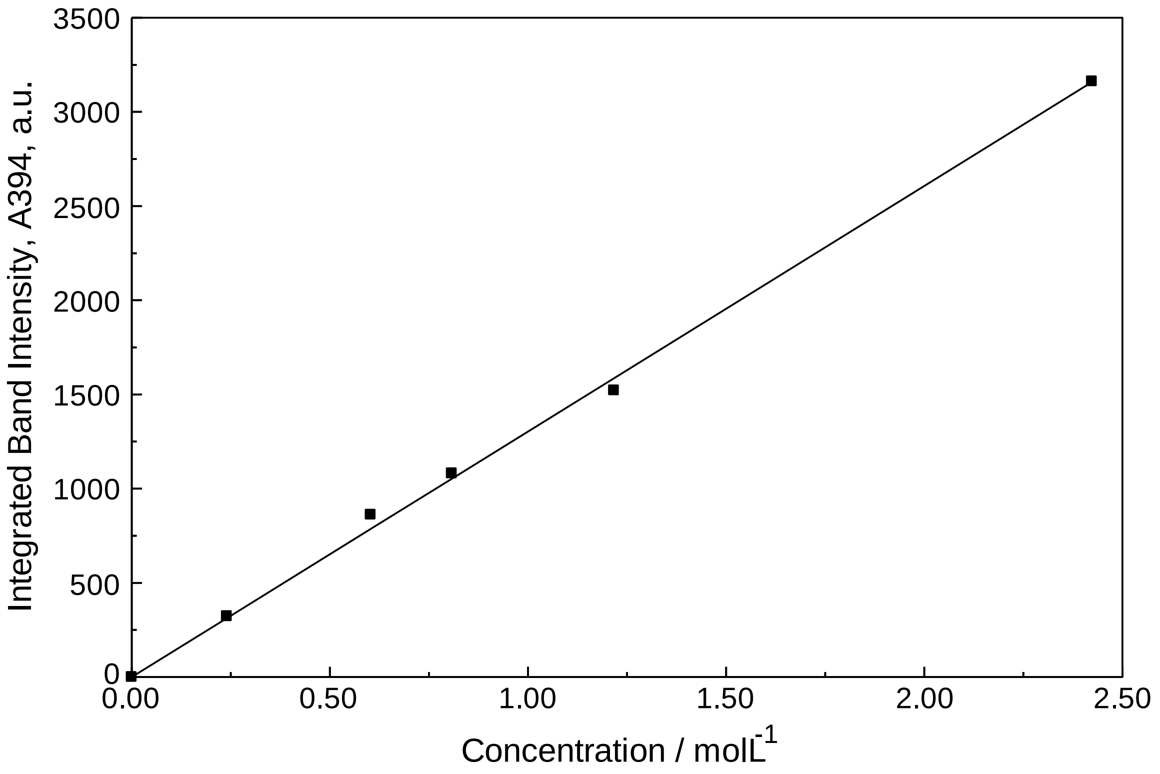

Figure S3. Integrated band intensity, A_{394}, of the symmetric stretching mode $\nu_1 YbO_8$ in arbitrary units as a function of the $Yb(ClO_4)_3$ solution concentration.

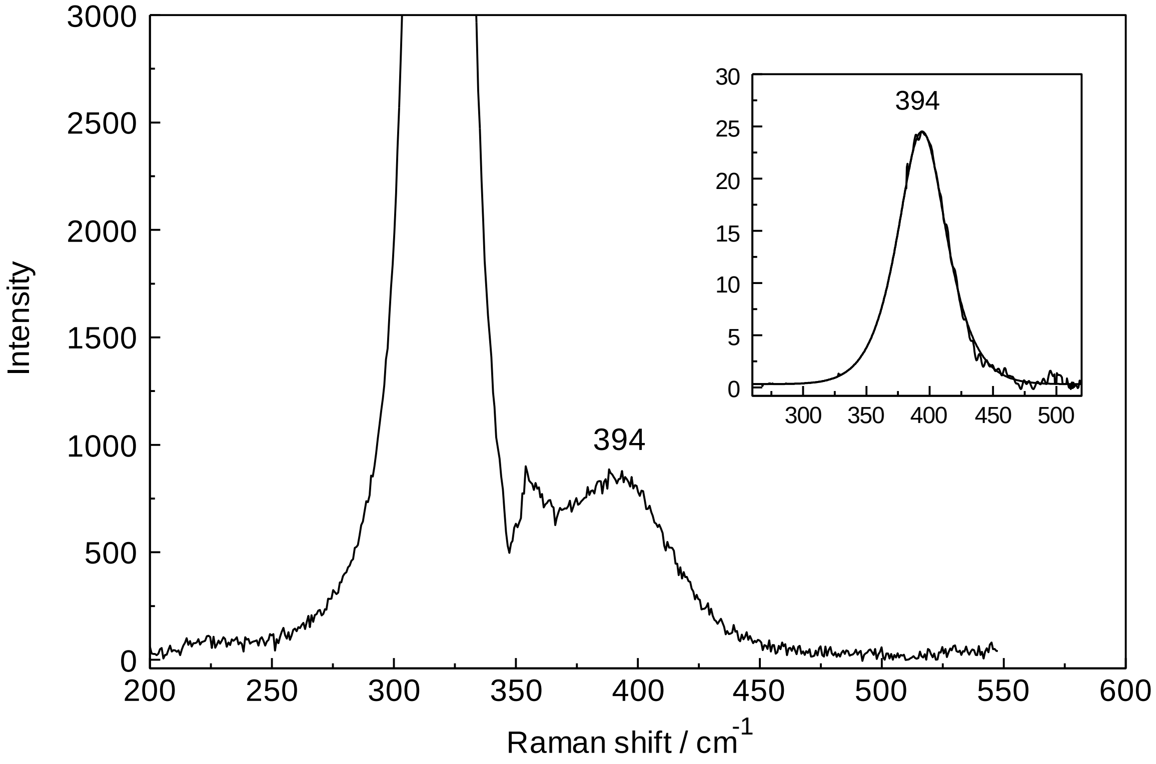

Figure S4. Isotropic Raman scattering profile (R-format) of a 1.25 $molL^{-1}$ $Yb(CF_3SO_3)_3$ solution. In addition to the strong triflate band at 319.5 cm^{-1} a very weak and relatively broad band appears at 394 cm^{-1} which is assigned to the totally symmetric stretching mode of YbO_8.

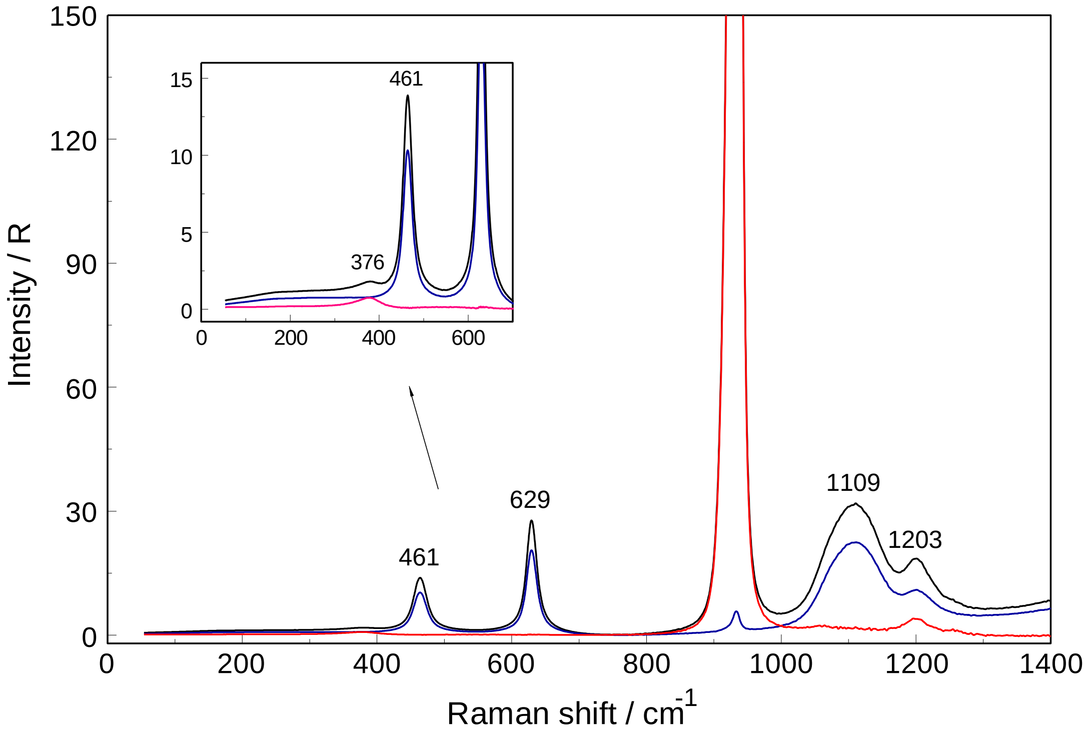

Figure S5. Raman scattering profiles of a 1.276 molL^{-1} $Yb(ClO_4)_3$ solution in heavy water in order to observe the isotope effect on the symmetric stretching mode of the hydrate respectively deuterated species by changing from $[Yb(H_2O)_8]^{3+}(H_2O)$ to $[Yb(D_2O)_8]^{3+}(D_2O)$. Shown are the isotropic scattering profile (red), the anisotropic one (blue) and the polarized scattering (black).

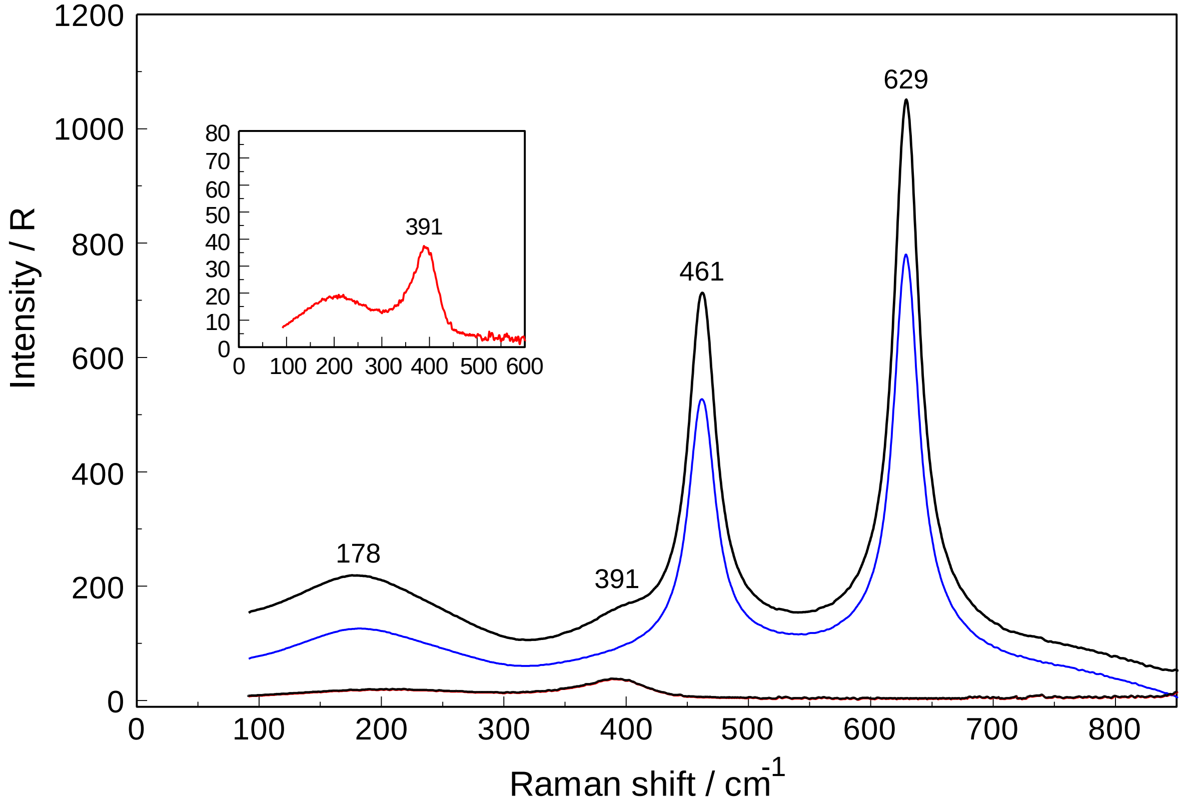

Figure S6. $Tm(ClO_4)_3$ solution at 0.315 $molL^{-1}$ (R_w = 226.6) in R-format (spectra from top to bottom: R_{VV}, R_{VH} and R_{iso}). The inset shows the R_{iso} spectrum in greater detail. Note the broad and weak ν_1TmO_8 stretching mode at 391 cm^{-1} (fwhh = 53 cm^{-1}) of the $[Tm(OH_2)_8]^{3+}$ species. The much larger, depolarized bands at 461 cm^{-1} and 629 cm^{-1} are the deformation modes of perchlorate, ClO_4^-(aq). Shown are the isotropic scattering profile (red), the anisotropic one (blue) and the polarized scattering (black).

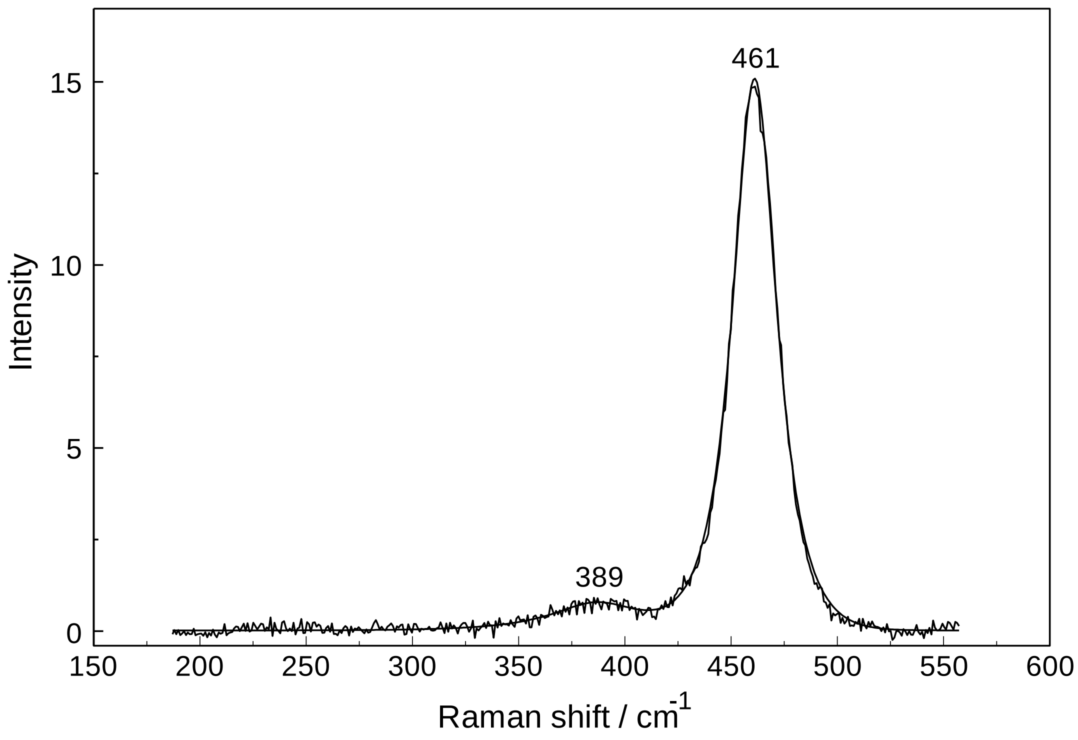

Figure S7. Polarized Raman scattering profile of an aqueous $Er(ClO_4)_3$ solution at 0.321 $molL^{-1}$. The weak and strongly polarized mode at 389 cm^{-1} is assigned to the Er-O symmetric stretching mode of $[Er(OH_2)_8]^{3+}$. Note the strong deformation mode, ν_2(e) ClO_4^-(aq) at 461 cm^{-1}.

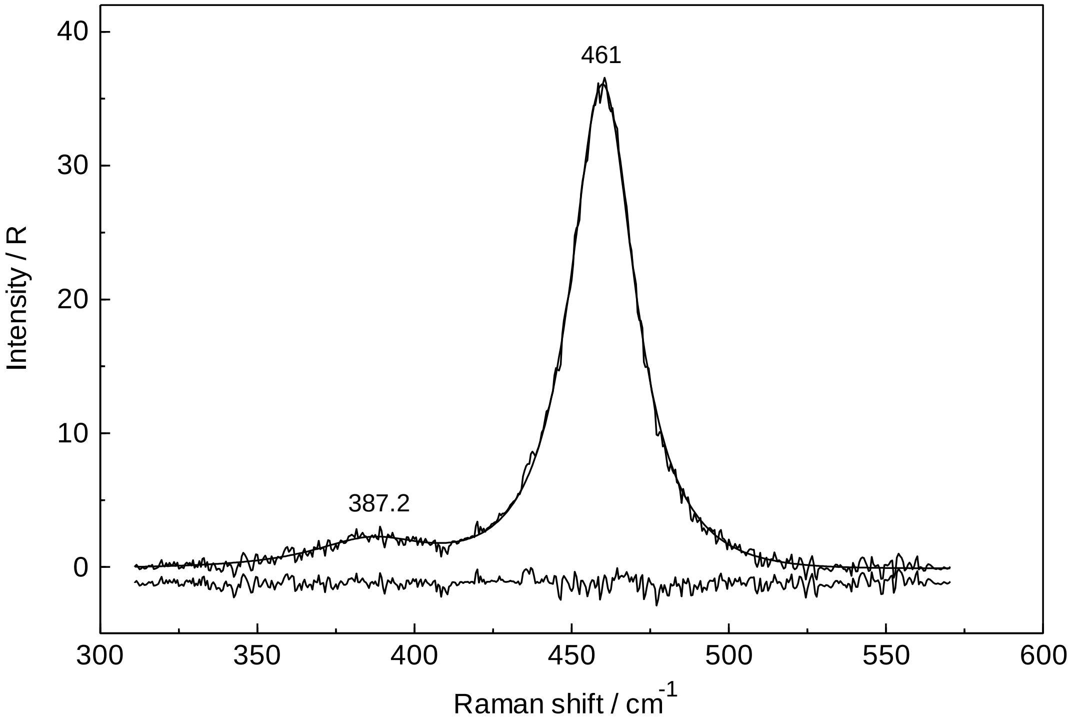

Figure S8. Polarized Raman scattering profile of an aqueous $Ho(ClO_4)_3$ solution at 0.240 molL^{-1}. The weak and strongly polarized mode at 387 cm^{-1} is assigned to the Ho-O symmetric stretching mode of $[Ho(OH_2)_8]^{3+}$. Note the strong deformation mode, ν_2(e) ClO_4^-(aq) at 461 cm^{-1}.

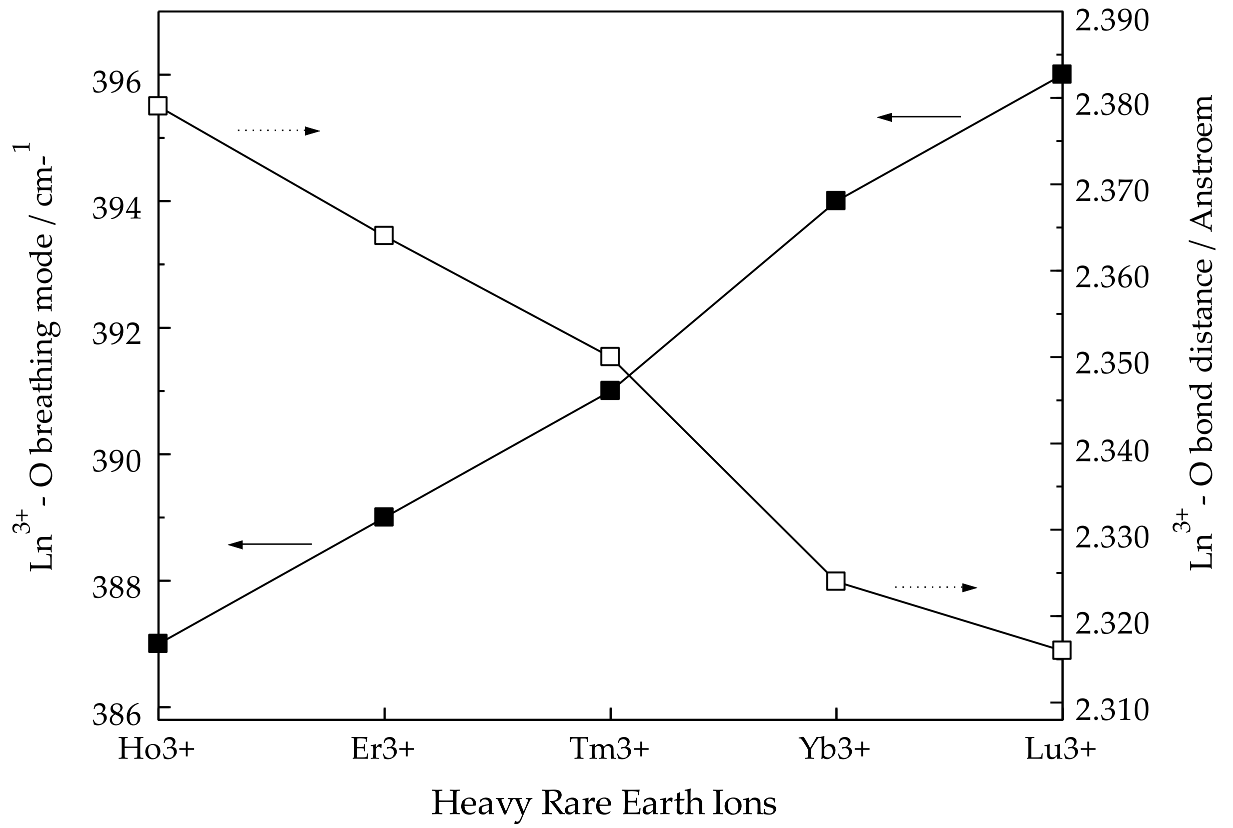

Figure S9. The heavy REE ions at the X-axis from left to right: Ho^{3+}, Er^{3+}, Tm^{3+}, Yb^{3+}, and Lu^{3+}. At the left Y-axis the peak positions of the corresponding breathing modes, ν_1 Ln-O are given and at the right Y-axis the Ln-O bond distances of the octahydrates, $[Ln(OH_2)_8]^{3+}$ [7].

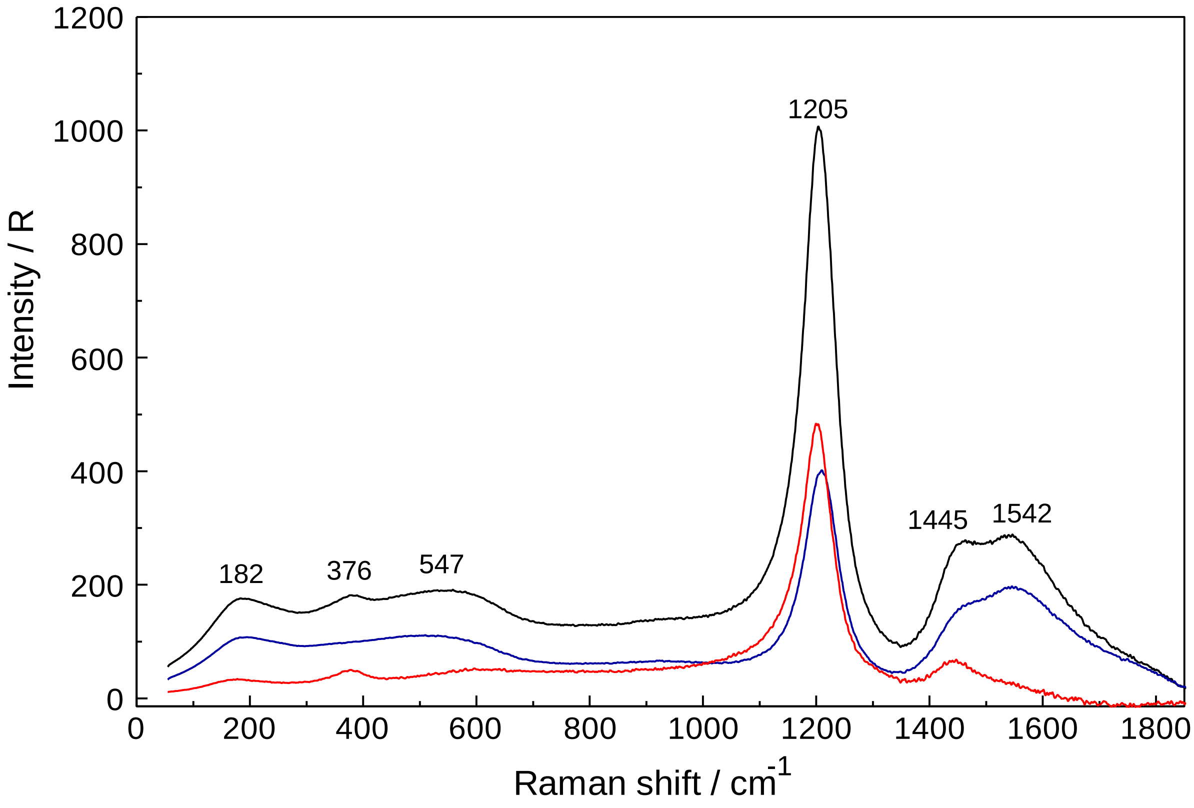

Figure S10. Raman scattering profiles in R-format (R_{VV} (black), R_{VH} (blue) and R_{iso} (red) scattering) of a 0.422 molL^{-1} $YbCl_3$ solutions in heavy water. The YbO_8 symmetric stretch of $[Yb(D_2O)_8]^{3+}(D_2O)$ (D_2O taken as point mass) is shifted to 376 cm^{-1} while the same mode in light water is observed at 394 cm^{-1} demonstrating the vibrational isotope effect on the symmetric stretching mode by changing from $[Yb(H_2O)_8]^{3+}(aq)$ to $[Yb(D_2O)_8]^{3+}(D_2O)$. Note furthermore the restricted translation band of the O-D···D unit at 182 cm^{-1}, the librational band at 547 cm^{-1} and the deformation mode ν_2 D-O-D at 1205 cm^{-1}. The mode at 1445 cm^{-1} stems from the small amount of HDO and the band at 1542 cm^{-1} is a combination band of D_2O.

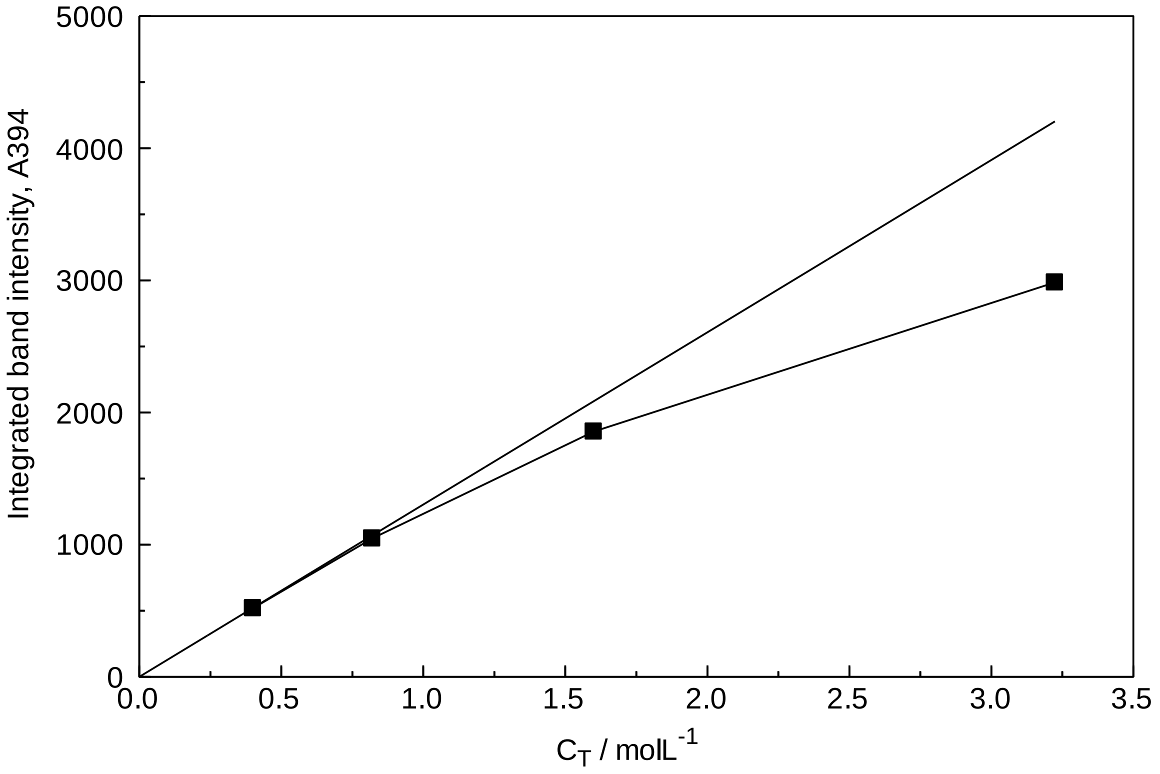

Figure S11. The linear dependence of the integrated band intensity, A_{394} as a function of the $Yb(ClO_4)_3$ concentration with A_{394}=1303.7*C_T (see Figure S4). The lower curve shows the integrated band intensity of the band at 394 cm^{-1} in $YbCl_3$ solutions (black squares). The solute concentration is denoted as C_T.
